# Efficacy and safety of fospropofol disodium sedation for same-day bidirectional endoscopy in elderly patients: protocol for a prospective, single-center, randomized, double-blind, non-inferiority trial

**DOI:** 10.3389/fphar.2024.1378081

**Published:** 2024-08-08

**Authors:** Zhe Zhao, Xiaogao Jin, Yong Li, Xiaofeng Wang, Yongchen Cui, Bing Zhang, Yu Kang, Guangming Zhang, Qinjun Chu, Junfeng Zhang

**Affiliations:** ^1^ Department of Geriatrics, Shanghai Sixth People’s Hospital Affiliated to Shanghai Jiaotong University School of Medicine, Shanghai, China; ^2^ Department of Anesthesiology, The Second Affiliated Hospital of Guangdong Medical University, Zhanjiang, Guangdong, China; ^3^ Department of Anesthesiology, Tongren Hospital, Shanghai Jiao Tong University School of Medicine, Shanghai, China; ^4^ Department of Anesthesiology, Shanghai Sixth People’s Hospital Affiliated to Shanghai Jiaotong University School of Medicine, Shanghai, China; ^5^ Department of Anesthesiology and Perioperative Medicine, Zhengzhou Central Hospital Affiliated to Zhengzhou University, Zhengzhou, Henan, China

**Keywords:** fospropofol, propofol, sedation, elderly, bidirectional endoscopy

## Abstract

**Introduction:**

Fospropofol disodium is a novel prodrug that has improved pharmacokinetic and pharmacodynamic properties when compared with propofol. This trial aims to compare the efficacy and safety of fospropofol *versus* propofol sedation for same-day bidirectional endoscopy in elderly patients.

**Methods and analysis:**

This is a prospective, single-center, double-blind, randomized, propofol-controlled, non-inferiority trial. A total of 256 patients aged 65 years or older, who are scheduled for same-day bidirectional endoscopy under sedation, will be randomly allocated, in a 1:1 ratio, to either fospropofol group or propofol group (n = 128 in each group). All patients will receive analgesic pre-treatment with sufentanil 5 μg. Two minutes later, an initial bolus dose of fospropofol 6.5 mg/kg or 1.5 mg/kg propofol and supplemental doses of fospropofol 1.6 mg/kg or 0.5 mg/kg propofol will be titrated as needed to achieve target sedation levels during the procedures. The primary outcome is the success rate of same-day bidirectional endoscopy. Secondary outcomes include the time to successful induction of sedation, duration, time to being fully alert, time to patient discharge, endoscopist satisfaction, patient satisfaction, and the top-up frequency and dosage of sedative medications. The safety endpoints consist of adverse events concerning cough reflex, gag reflexes, body movement, muscular tremor, and pain on injection. Sedation-related AEs, including episodes of desaturation, severe desaturation (SpO_2_ < 90%), hypotension, severe hypotension (decrease in MBP ≥30% of baseline), and bradycardia, will also be recorded. Data will be analyzed on an intention-to-treat basis.

**Discussion:**

We hypothesize that the efficacy and safety of fospropofol sedation for elderly patients undergoing same-visit bidirectional endoscopy will not be inferior to that of propofol. Our findings will potentially provide a new sedation regimen for same-visit bidirectional endoscopy in elderly patients.

**Clinical Trial Registration:**

clinicaltrials.gov, identifier NCT02875639

## 1 Introduction

With life expectancy rising around the world, the incidence of gastrointestinal diseases increases in parallel with the aging of population. Research statistics indicate that elderly people account for a high proportion of all newly diagnosed gastrointestinal malignancies (Hall et al., 2005). Gastrointestinal endoscopy is an effective diagnostic method for gastrointestinal diseases. Same-day bidirectional endoscopy (esophagogastroduodenoscopy and colonoscopy) has become increasingly common due to its cost-effectiveness and ability to enhance decision-making (Urquhart et al., 2009). Recent studies have shown that the optimal sequence for patients undergoing same-day bidirectional endoscopy under sedation is esophagogastroduodenoscopy followed by colonoscopy (Laoveeravat et al., 2020).

Moderate sedation is necessary during gastrointestinal endoscopy to reduce the patient’s memory of the event, make them more comfortable and less anxious, and significantly improve the procedure outcome (Early et al., 2018; Zhou et al., 2021). Though the types of medications used for sedation in elderly patients are not different from those in younger patients, it is important to be aware of the increased sensitivity of this population to sedative medications (Chandrasekhara et al., 2013). Elderly patients are at increased risk for adverse events, such as hypoxemia, hypotension, arrhythmias, and esophageal reflux during the gastrointestinal endoscopy (Laanani et al., 2019; Shimizu et al., 2021). Therefore, clinicians should pay more attention when administering sedative medications to elderly patients undergoing gastrointestinal endoscopy. Over the long term, propofol is the most commonly used sedative agent for gastrointestinal endoscopy due to its rapid onset of action and fast recovery (Zhang et al., 2018). Propofol combined with opioids has become the most common regimen of sedation in China (Zhou et al., 2021). However, due to its narrow therapeutic window, propofol poses high risks of adverse events such as respiratory depression, hypotension, and pain on injection, especially in elderly patients (Shimizu et al., 2021).

Fospropofol disodium for injection (manufactured by Yichang Human well Pharmaceutical Co., Ltd., Hubei, P. R. China) is a novel water-soluble prodrug of propofol. It has a unique pharmacokinetic and pharmacodynamic profile compared to lipid propofol (Wu et al., 2021). As a prodrug of propofol, fospropofol’s pharmacologic activity results from its breakdown by alkaline phosphatase and subsequent release of propofol (Liu et al., 2020). Compared to propofol lipid emulsion, it exhibits a longer time to peak clinical effect and a more prolonged action, resulting in smoother hemodynamic and respiratory depression in patients (Abdelmalak et al., 2012). Additionally, it does not cause a burning sensation upon IV administration, which is a common side effect of propofol (Garnock-Jones and Scott, 2010). The available evidence suggests that fospropofol sedation is effective for patients undergoing esophagogastroscopy, colonoscopy, and flexible bronchoscopy (Cohen et al., 2010; Gan et al., 2010; Bergese et al., 2013; Wu et al., 2021). Despite these promising findings, the optimal sedation regimen for fospropofol to facilitate same-day bidirectional endoscopic procedures remains unclear. Moreover, it is worth noting that most studies have been conducted on populations with a wide age range and have not investigated the safety of fospropofol in elderly patients (Abdelmalak et al., 2012). As a result, we designed this study to evaluate the efficacy and safety of fospropofol in comparison with propofol for sedating elderly patients undergoing same-day bidirectional endoscopy.

## 2 Materials and methods

### 2.1 Study design

This is a prospective, single-center, double-blind, randomized, propofol-controlled, non-inferiority trial, which will be carried out at Tongren Hospital, School of Medicine, Shanghai Jiao Tong University, Shanghai, China. This trial protocol was approved by the Medical Ethics Committee of Tongren Hospital, School of Medicine, Shanghai Jiao Tong University (Approval No. 2023–035) and registered at the ClinicalTrials.gov (Identifier: NCT06251999) on 21 January 2024. The implementation of this trial will be in accordance with the Declaration of Helsinki. The protocol follows the guidelines of Standard Protocol Items: Recommendations for Interventional Trials (SPIRIT) (Chan et al., 2013).

### 2.2 Participants

**FIGURE 1:**
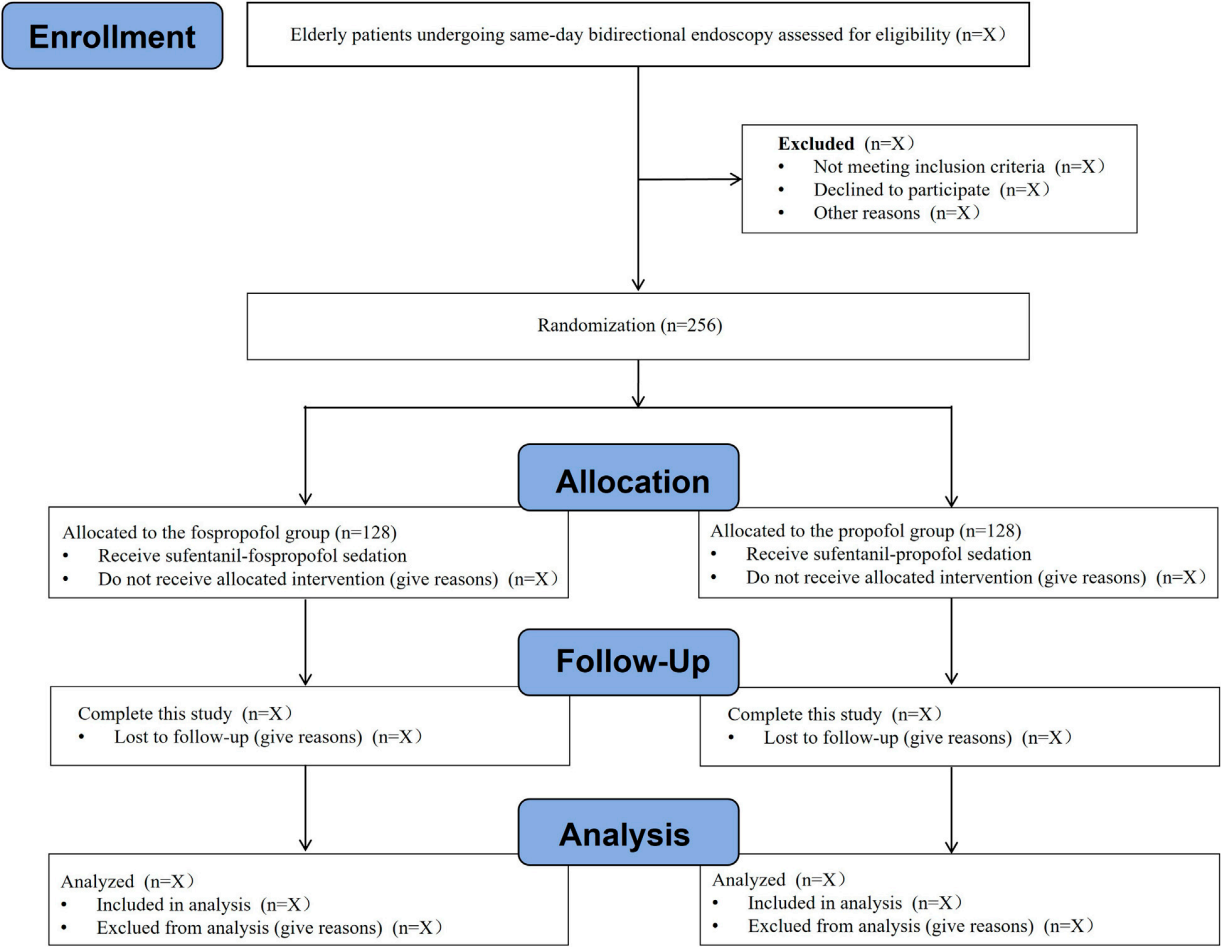
The flow chart of this study.

An independent investigator, who is not involved in the subsequent study, screens the admission records for recruitment of eligible patients. Written informed consent will be obtained from the patient or their representative before each patient performed any procedure. At any time during this study, patients can withdraw their consent without any consequence. A total of 256 eligible patients aged 65 years or older, who are scheduled for same-day bidirectional endoscopy under sedation, will be randomly allocated to either fospropofol group or propofol group at a ratio of 1:1 (n = 128 in each group). The flow chart of this study is presented in Figure 1.

#### 2.2.1 Inclusion criteria

The inclusion criteria are as follows.

1) 65 years or older; 2) American Society of Anesthesiologists (ASA) physical status I to III; 3) body mass index (BMI) 18–30 kg/m^2^, and 4) scheduled for same-day bidirectional endoscopy under sedation.

#### 2.2.2 Exclusion criteria

The exclusion criteria are as follows.

1) severe cardiovascular, pulmonary, renal, or hepatic disease; 2) previous hypotension (systolic blood pressure ≤90 mmHg), bradycardia (heart rate <50 beats/min), or hypoxemia (SpO2 <90%); 3) neurocognitive or psychiatric disorders; 4) contraindications to gastroscopy (gastric retention, long-term aspirin administration, etc.); 5) hypersensitivity to study medications; 6) drug or alcohol abuse; 7) definite upper respiratory tract infection; or 8) refusal to participation.

### 2.3 Randomization and blinding

Eligible patients will be randomly assigned to either the fospropofol group or the propofol group at a 1:1 ratio using a computer-generated sequence. Group assignments are placed into opaque, sealed, consecutively numbered envelopes by staff who is not involved in the trial. The envelopes will be opened just before the gastrointestinal endoscopy by a certified registered anesthetist who also prepares the drugs in coded syringes according to the order number indicating the group of assignment, and she/he is not associated with patient management or data collection and analysis. Patients, endoscopists, anesthesiologist, peri-procedural care providers, and outcome observers are blinded to group assignment until the end of final analysis.

### 2.4 Study procedures

On the day of the gastrointestinal endoscopy, the patient will be given an intravenous infusion channel in the waiting room. After admission to the endoscopy room, all patients will be placed in a left lateral decubitus position and monitored according to our hospital standards including real-time monitoring of electrocardiogram (ECG), peripheral blood oxygen saturation (SpO_2_), end-tidal carbon dioxide (EtCO_2_), non-invasive blood pressure (NIBP), and respiratory rate (RespR) measured every 5 min. Supplemental oxygen will be given via a transparent nasal cannula at 5 L/min throughout the procedure.

The patients will receive intravenous sedation regimen according to previous literature (Gan et al., 2010; Bergese et al., 2013), administered by the same anesthesiologist unaware of patient allocation group. All patients receive analgesic pre-treatment with sufentanil 5 μg via an intravenous infusion channel. It is recommended that patients receive only one additional dose of sufentanil 2.5 μg if the patient continues to experience pain during the procedure; however, additional doses of sufentanil (given at least 10 min after the previous dose) are allowed. After 2 min, an initial bolus dose of fospropofol 6.5 mg/kg or 1.5 mg/kg propofol and supplemental doses of fospropofol 1.6 mg/kg or 0.5 mg/kg propofol will be administered as needed to achieve a Modified Observer’s Assessment of Alertness/Sedation (MOAA/S) score ≤4 according to random group, allowing the physician to start the procedure. The bidirectional endoscopic procedures will be performed by experienced professional endoscopic teams in an esophagogastroduodenoscopy-colonoscopy sequence (Cao et al., 2017; Laoveeravat et al., 2020). This dosing schedule is also used to maintain adequate sedation levels during the procedure if the patient has a MOAA/S score ≥4 and shows purposeful movement. As deep levels of sedation (MOAA/S scores of 0 and 1) may be associated with an increased risk of respiratory compromise, additional doses of fospropofol or propofol are not administered to patients who had a MOAA/S score below four or who is not show purposeful movement during the sedation phase. If the patient sufferes from respiratory depression (SpO_2_ <90% for >10 s), the essential respiratory support such as chin/jaw lifting or assisted mask ventilation will be provided immediately until SpO_2_ returns to normal. In case of hypotension (MAP <70 mmHg or the decline reached 20% of the basal value) lasting more than 1 min, rehydration and intravenous injection of 50 µg phenylephrine will be administered. If bradycardia (HR < 50 beats/min) occurred, 0.5 mg atropine will be given intravenously.

Upon the completion of endoscopic procedures, the anesthesiologist will assess the patient’s consciousness and hemodynamic data, and then transfer the patient to the post-anesthesia care unit (PACU) for postoperative care. All patients will stay in the recovery room for at least 30 min. The modified Aldrete Score is used to assess the overall recovery of patients. Patients are allowed to be discharged if a modified Aldrete score of nine or more is identified (Laoveeravat et al., 2020). The initial dose of sedative medication and the number and dose of supplemental doses given will be recorded, and the total amount of sedative medication administered to the patient is calculated. The schedules of patient enrollment, study interventions, and outcome assessments will be in accordance with the SPIRIT statement (Table 1).

**TABLE 1:** Schedule of enrollment, interventions, and assessments.

Timepoint	Study period						
	Enrollment	Allocation	Post-allocation				Close-out
	Anesthesia clinic visit	Prior to sedation	During sedation	Sedation emergence	15 min in recovery room	30 min in recovery room	Hispital discharge
Enrollment							
Eligibility criteria	×						
Exclusion criteria	×						
Written informed consent	×						
Demographic data	×						
Baseline characteristics	×						
Randomization		×					
Allocation		×					
Interventions							
Propofol		×					
Fospropofol		×					
Assessments							
The success rate				×			
Time to successful induction			×				
Time to being fully alert				×			
Time to discharge							×
Total dosage of experimental drugs				×			
Total dosage of sufentanil				×			
Total times of top-up dosing				×			
Top-up dosing times of sufentanil				×			
Endoscopist satisfaction			×				
Patient satisfaction							×
Cough reflex			×				
Gag reflexes			×				
Body movement			×				
Muscular tremor			×				
Injection pain			×				
Nausea and vomiting				×	×	×	×
Paresthesia				×	×	×	×
Pruritus				×	×	×	×
Apnea			×	×	×	×	×
Hypoxia events			×	×	×	×	×
Hypotension events			×	×	×	×	×
Bradycardia			×	×	×	×	×

According to SPIRIT, 2013 statement of defining standard protocol items for clinical trials (Chan et al., 2013).

### 2.5 Efficacy assessments

#### 2.5.1 Primary efficacy outcomes

The success rate of the gastrointestinal endoscopy will be assessed according to the following criteria (Luo et al., 2022; Zhong et al., 2023): (1) completion of the gastrointestinal endoscopy; (2) no need for rescue sedative/anesthetic, which means the top-up doses of the experimental drugs will be administered no more than five times within any 15-min window from the initial administration to the end of the procedure.

#### 2.5.2 Secondary efficacy outcomes

(1) Time to successful induction of anesthesia/sedation, defined as the time from the start of drug administration to the achievement of a MOAA/S score ≤1; (2) time to being fully alert, defined as the time from gastrointestinal endoscopy extraction or/and the time from the last drug administration to a MOAA/S score of five on three consecutive measurements; (3) time to discharge, defined as the time from the gastrointestinal endoscopy extraction or/and the time from the last drug administration to the initial occurrence of three consecutive Aldrete measurements of 9; (4) the top-up frequency and dosage of the study medications and sufentanil; (5) anesthesia/sedation satisfaction scores of the patients and endoscopists collected when the patients are ready for discharge.

### 2.6 Safety assessments

Safety will be assessed by the rate of occurrence of adverse events (AEs). AEs are evaluated for frequency, severity, association to the study drug, relationship to procedure, and outcome. The severity of AEs is graded based on the Common Terminology Criteria for Adverse Events (CTCAEs, version 5.0). The causal relationship of an AE to the investigated drug (fospropofol or propofol) will be assessed by the investigator using the classifications shown in Table 2 according to previous literature (Luo et al., 2022). AEs are considered severe if they affects a patient’s daily function or threatens their life. If cough reflex, gag reflexes, body movement, muscular tremor, pain on injection, or other reactions caused by operation irritation will be observed in patients, they are recorded as operational reactions of gastrointestinal endoscopy. Sedation-related AEs, including hypoxia, hypotension, bradycardia, and prolonged sedation, are evaluated from the time of administration of bolus doses of the study drug until the discharge of patients. Hypoxia is defined as pulse oxygen saturation (SpO_2_) < 90% lasting for >30 s; hypotension is defined as systolic blood pressure (SBP) < 90 mmHg or a decrease of 20% from baseline lasting for >2 min; bradycardia is defined as heart rate (HR) < 50 beats/min and lasting for >2 min. Prolonged sedation is defined as a MOAA/S score ≤4 for more than 30 min after the end of the colonoscopy.

**TABLE 2:** Classification of AEs potentially associated with fospropofol or propofol.

Indicator	Definitely related	Probably related	Possibly related	Possibly not related	Not related
Reasonable time sequence	Yes	Yes	Yes	Yes	Yes
Belongs to the type of reaction known to be produced by the study drug	Yes	Yes	Yes	No	No
Reaction may be improved after discontinuation of the study drug	Yes	Yes	Yes or No	Yes or No	Yes or No
Reaction may re-occur with medication	Yes	?	?	?	No
There is another explanation for the reaction	No	No	Yes	Yes	Yes

### 2.7 Data collection

Demographic data [including age, sex, race, height, weight, and body mass index (BMI)] and baseline characteristics (including comorbidities, preoperative medications, baseline MAP and HR values, and ASA status) will be collected prior to the procedures. The primary and secondary outcome measures and other perioperative data will be documented by a trained independent investigator blinded to group assignment. All raw data will be recorded on the Case Report Forms. The lead investigator will be responsible for the completeness and accuracy of data.

### 2.8 Safety monitoring

Propofol is widely used for sedation in various clinical settings, and emerging literature has demonstrated the use of fospropofol in patients undergoing gastrointestinal endoscopy or other clinical procedures (Cohen et al., 2010; Candiotti et al., 2011; Abdelmalak et al., 2012; Bergese et al., 2013; Wu et al., 2021). Thus, serious adverse events related to the interventions in this trial are less likely to occur. However, in case of a serious adverse event, the attending anesthesiologist will provide immediate clinical management and such an event should be reported to the principal investigator and the Institutional Review Board. They will decide whether the unmasking process of group allocation should be done.

### 2.9 Sample size evaluation

The study is designed as a non-inferiority trial with a non-inferiority margin (δ) of 8% in terms of the success rate (Luo et al., 2022; Zhong et al., 2023). At a power of 80%, a one-sided alpha of 0.025, and assuming that the success rate of gastrointestinal endoscopy after fospropofol or propofol administration is both 95%, a total of 234 patients are calculated using PASS software (version 2021, NCSS, United States). Together with an anticipated drop-out rate of 10%, a total of 256 patients (128 patients in each group) will be required for this trial.

### 2.10 Data management and statistical analysis

Statistical analysis in this trial will be performed using SPSS 25 (SPSS Inc., Chicago, Illinois, United States). Continuous variables are presented as the mean ± standard deviation (SD) or median with the upper and lower quartiles, whereas categorical variables are presented as frequencies and percentages. A P-value <0.05 is considered statistically significant. For the primary efficacy outcome, the difference in gastrointestinal endoscopy success rate between fospropofol and propofol groups and the corresponding two-sided 95% confidence intervals (CIs) will be calculated. Non-inferiority between fospropofol and propofol is concluded if the lower limit of the two-sided 95% CI of the difference in success rate between fospropofol and propofol groups exceeds the non-inferiority margin (−8%). The success rates of gastrointestinal endoscopy will be compared between the fospropofol and propofol groups using the Chi-square test or Fisher’s exact test. The Shapiro-Wilk’s test will be used to assess the data distribution. For comparison of secondary efficacy outcomes, the Cochran–Mantel–Haenszel (CMH) test or Fisher’s exact test will be used to compare the categorical variables between two groups, while a t-test (normal distribution) or Mann-Whitney U test (non-normal distribution) is used to compare continuous variables, as appropriate. The incidence of AEs and drug-related AEs between the two groups will be compared using a chi-square test or Fisher’s exact test. In addition, the further interaction analysis across the subgroups, including age (65–79 years vs. ≥ 80 years), history of cardiovascular, renal, hepatic, and pulmonary diseases (yes vs. no), as well as interventional therapeutic endoscopic procedures (yes vs. no), will be carried out using a logistic regression model to adjust the study outcomes for above-mentioned potential confounding factors. All randomly assigned patients who receive at least one dose of the study medication will be included in the full analysis set (FAS) according to the intention-to-treat (ITT) principle, and at least one evaluable efficacy outcome is used for the analysis of all efficacy outcomes and baseline characteristics of patients. The safety set (SS), which includes all patients based on the “as treated” principle, will be used for the analysis of safety outcomes. Since the anticipated protocol violation will be uncommon and missing data will be supposed to be missing at random, the interim analysis or imputation for missing data are not performed in this trial.

## 3 Discussion

Over the past decade, the volume of gastrointestinal endoscopic procedures has increased 10-fold (Xin et al., 2022; Song et al., 2023). Many patients now undergo esophagogastroduodenoscopy and colonoscopy during the same hospital visit, commonly known as same-visit bidirectional endoscopy. Same-day bidirectional endoscopy has been increasingly implemented because it reduces costs and facilitates decision-making for other interventions or treatments needed by patients (Laoveeravat et al., 2020). To improve patient comfort and facilitate these procedures, the use of sedation has been increasingly requested by patients (Dossa et al., 2021). Propofol is a sedative agent commonly used for sedation in gastrointestinal endoscopy (Grocott, 2018; Zhang et al., 2018). Although its pharmacological properties render propofol an almost ideal drug to achieve and maintain the desired level of sedation in gastrointestinal procedures (Stogiannou et al., 2018), it is not without its drawbacks, as adverse events such as respiratory depression, hypotension, and pain on injection have been reported (Sneyd et al., 2022). In previous studies of patients undergoing gastrointestinal endoscopy with propofol sedation, the incidence of desaturation and hypotension events was approximately 30% (Chiang et al., 2013; Eberl et al., 2020). In an analysis of national claims databases in France, Laanani et al. found that SAEs related to screening and diagnostic colonoscopies were more frequent in older patients, particularly those with comorbidities (Laanani et al., 2019). Furthermore, same-visit bidirectional endoscopy is a more complex and prolonged endoscopic procedure compared to a single either esophagogastroduodenoscopy or colonoscopy. Therefore, special considerations should be given for elderly patients received same-visit bidirectional endoscopy. Currently, the best sedation regimen to facilitate a same-visit bidirectional endoscopy in an effective, safe and satisfactory manner for both patient and endoscopist is still not clear (Song et al., 2023).

Fospropofol is a water-soluble, phosphate ester prodrug, which can be metabolized by alkaline phosphatases to release liberated propofol after intravenous administration (Garnock-Jones and Scott, 2010). The released propofol from fospropofol binds to the γ-aminobutyric acid (GABA) receptor and thereby potentiates GABA-inhibitory synaptic currents to induce sedative effects (Garnock-Jones and Scott, 2010). A study of its pharmacodynamic/pharmacokinetic relationship in 12 healthy volunteers found that an intravenous bolus 10 mg/kg dose of fospropofola achieved propofol plasma maximum concentration of 2.2 mg/mLafter 8 (range 4–13) minutes, and reached the minimum mean MOAA/S score of 1.2 after 7 (1–15) minutes. Participants in this study completely recovered from sedation after 21–45 min following fospropofol administration (Bengalorkar et al., 2011). As a new sedative, fospropofol is expected to have the same sedative efficacy as propofol in elderly patients undergoing same-day bidirectional endoscopy, with a lower incidence of adverse reactions caused by hemodynamic and respiratory depression than that of propofol. On bassi of a phase II dose-response trial in patients undergoing colonoscopy, fospropofol 6.5 mg/kg was determined as the ideal dose, as no recorded episodes of deep sedation occurred during procedures under this dose (Cohen, 2008). In addition, both physicians and patients mostly preferred for fospropofol 6.5 mg/kg over the other doses (Cohen, 2008). In two clinical trials, fospropofol was shown to be safe and well tolerated at an initial dose of 6.5 mg/kg intravenously with supplemental doses as moderate sedation for elderly, obese, and high-risk patients in minor surgical procedures (Gan et al., 2010; Bergese et al., 2013). Fospropofol disodium for injection is a prodrug that is metabolized into propofol to produce a general anesthetic effect when administered intravenously. A phase 3 trial demonstrated that fospropofol disodium was not inferior to propofol for general anesthesia induction in American Society of Anesthesiologists (ASA) physical status I-II adult patients undergoing elective surgery and reduced the incidence of pain at the injection site (Wu et al., 2021). In addition, fospropofol’s slower onset should have less impact on patient’s hemodynamics compared to propfol, although it may potentially slow patient recovery (Gan et al., 2010). In clinical conditions where a more prolonged effect is desired and immediate onset of action is less important, fospropofol may have advantages over propofol. A pilot study has shown that fospropofol, administered in either an infusion/bolus or infusion-only regimen, is tolerable and effective for the short-term induction and maintenance of sedation in mechanically ventilated intensive care unit patients (Candiotti et al., 2011). These findings have prompted to exploration of fospropofol in providing sedation for elderly patients undergoing same-day bidirectional endoscopic procedures. However, the role of fospropofol sedation for same-day bidirectional endoscopy in elderly patients has not been fully determined. To our knowledge, this will be the first randomized controlled trial to assess the efficacy and safety of fospropofol sedation for same-day bidirectional endoscopy in elderly patients. In our patients, an initial bolus dose of fospropofol 6.5 mg/kg in combination with sufentanil 5 μg will be used for sedation induction, followed by supplemental doses of fospropofol 1.6 mg/kg as needed to achieve target sedation levels. This strategy will minimize the risk of oversedation and associated complications. Moreover, several confounding factors, which can influence the study outcomes, must be appropriately managed in this study to ensure that the reliable conclusions are obtaine. Subgroup analyses of the efficacy and safety assessments will be performed on basis of age, interventional therapeutic endoscopic procedures, history of cardiovascular, renal, hepatic, and pulmonary diseases.

Several limitations must be pointed out in this study. First, our study will specifically include patients aged over 65 years with an ASA physical status I to III and a BMI between 18 and 30 kg/m^2^. Although these inclusion criteria will allow us to evaluate the anesthetic effect of fospropofol in this specific patient population, whether the conclusion from our study can be fully extrapolated to other elderly patients with higher ASA classifications or individuals with obesity warrants further investigation. Second, this trial don’t include different doses in the fospropofol group. We only evaluate 6.5 mg/kg fospropofol in combination with sufentanil for same-day bidirectional endoscopy in elderly patients because we intend to assess the efficacy and safety of fospropofol under the conventional effective dosage on basis of previous studies (Cohen, 2008; Bergese et al., 2013). The next step will be to investigate the dose-response relationship to establish optimal dosage of fospropofol sedation for elderly patients undergoing same-visit bidirectional endoscopic procedures. Finally, this study was a relative small sample, single center trial, and a prospective, multicenter trial with a larger sample size are needed to validate the preliminary results of this study. Despite these limitations, our study will provide valuable insights into the potential benefits of forpropofol-based sedation for same-day bidirectional endoscopy in elderly patients. Further research in larger sample size will be essential to determine optimal dosing regimens and extend the applicability of fospropofol to the broader patient populations undergoing same-visit bidirectional endoscopic procedures.

In conclusion, this prospective, randomized, double-blind, non-inferiority trial will verify the efficacy and safety of fospropofol sedation for elderly patients undergoing same-visit bidirectional endoscopy and confirm whether it is inferior to propofol. We believe that fospropofol represents a new option for sedation of elderly patients during same-visit bidirectional endoscopic procedures.
